# Molecular and phenotypic characterization of *Colletotrichum* species associated with anthracnose disease in peppers from Sichuan Province, China

**DOI:** 10.1038/srep32761

**Published:** 2016-09-09

**Authors:** Fangling Liu, Guiting Tang, Xiaojuan Zheng, Ying Li, Xiaofang Sun, Xiaobo Qi, You Zhou, Jing Xu, Huabao Chen, Xiaoli Chang, Sirong Zhang, Guoshu Gong

**Affiliations:** 1 College of Agronomy & Key Laboratory for Major Crop Diseases, Sichuan Agricultural University, Chengdu, 611130, P.R. China; 2 College of Environmental Sciences, Sichuan Agricultural University, Chengdu, 611130, P.R. China

## Abstract

The anthracnose caused by *Colletotrichum* species is an important disease that primarily causes fruit rot in pepper. Eighty-eight strains representing seven species of *Colletotrichum* were obtained from rotten pepper fruits in Sichuan Province, China, and characterized according to morphology and the glyceraldehyde-3-phosphate dehydrogenase (GAPDH) sequence. Fifty-two strains were chosen for identification by phylogenetic analyses of multi-locus sequences, including the nuclear ribosomal internal transcribed spacer (ITS) region and the β-tubulin (TUB2), actin (ACT), calmodulin (CAL) and GAPDH genes. Based on the combined datasets, the 88 strains were identified as *Colletotrichum gloeosporioides*, *C. siamense*, *C. fructicola*, *C. truncatum*, *C. scovillei*, and *C. brevisporum*, and one new species was detected, described as *Colletotrichum sichuanensis*. Notably, *C. siamense* and *C. scovillei* were recorded for the first time as the causes of anthracnose in peppers in China. In addition, with the exception of *C. truncatum*, this is the first report of all of the other *Colletotrichum* species studied in pepper from Sichuan. The fungal species were all non-host-specific, as the isolates were able to infect not only *Capsicum* spp. but also *Pyrus pyrifolia* in pathogenicity tests. These findings suggest that the fungal species associated with anthracnose in pepper may inoculate other hosts as initial inoculum.

Pepper (*Capsicum annuum*), an important fruit that is also used as a spice, is rich in vitamins, capsaicin and capsochrome. One of the primary pepper-growing provinces in China is Sichuan Province, where the crop is cultivated over an area of approximately 70 thousand hm^2^, with approximately 1,000 thousand tons of annual output.

*Colletotrichum* is an important pathogenic genus worldwide. These fungi cause disease symptoms that are generally known as anthracnose in a wide range of vegetables, fruits and other crops^1^. In pepper, anthracnose is a destructive disease caused by a complex of *Colletotrichum* species that causes extensive yield losses at both the pre- and post-harvest stages during warm and rainy seasons^2^.

Anthracnose in pepper is associated with at least eleven *Colletotrichum* species, including *C. truncatum*^3^,^4^,^5^,^6^, *C. gloeosporioides*^6^,^7^,^8^,^9^, *C. acutatum*^6^,^10^,^11^, *C. coccodes*^12^,^13^,^14^,^15^, *C. fructicola*^7^,^16^,^17^, *C. siamense*^17^,^18^, *C. dematium*^14^, *C. boninense*^19^,^20^, *C. brevisporum*, *C. cliviae*^7^, and *C. scovillei*^21^. Eight of these species have been reported in China, whereas *C. siamense*, *C. dematium* and *C. scovillei* have not. To date, only three species (*C. acutatum*^22^, *C. truncatum*^5^ and *C. boninense*^19^) have been reported in Sichuan Province, although previous studies have not fully investigated the *Colletotrichum* species associated with pepper anthracnose in this province.

*Colletotrichum gloeosporioides* is a species complex that was formerly regarded as a cosmopolitan species that infects various hosts, including pepper; however, it might have been misidentified as the causative agent. For example, Phoulivong *et al*.^16^ failed to isolate *C. gloeosporioides* sensu stricto from tropical fruits, although *C. gloeosporioides* was previously thought to be the cause of tropical fruit rot. Similarly, Lima *et al*.^23^ found that none of the strains isolated from mango (a tropical fruit) belonged to *C. gloeosporioides* sensu stricto; instead, phylogenetic analysis revealed that most of the strains belonged to the ‘gloeosporioides’ complex. Although the nuclear ribosomal internal transcribed spacer (ITS) region is the most commonly used region for differentiating fungi^24^, it has also been widely acknowledged that this region cannot fully differentiate among *Colletotrichum* species^18^,^25^,^26^,^27^. Multi-locus phylogeny is broadly applied for identifying *Colletotrichum* spp. Weir *et al*.^18^ have suggested that the *C. gloeosporioides* complex consists of 23 taxa, according to multi-locus phylogeny. Several new species have also been described on the basis of multi-locus phylogeny, e.g., *C. anthrisci*, *C. liriopes*, *C. rusci* and *C. verruculosum*^3^; *C. bletillum*, *C. caudasporum*, *C. duyunensis*, *C. endophytum*, *C. excelsum-altitudum*, *C. guizhouensis* and *C. ochracea*^24^; *C. asianum*, *C. fructicola* and *C. siamense*^26^; *C. cliviae*, *C. hippeastri* and *C. hymenocallidis*^27^; *C. corchorum-capsularis*^28^; and *C. endophytica*^29^.

Despite several reports of *Colletotrichum* species in pepper from limited collection areas^5^,^19^,^22^, little is known about the association of these species with pepper in Sichuan Province, China. Further, it remains unclear whether all of the species isolated from pepper are equally pathogenic and host specific.

The objective of this study was to characterize the *Colletotrichum* species associated with anthracnose in pepper from different geographic areas of Sichuan Province, China, according to morphological, multi-locus phylogenetic and pathogenic characteristics.

## Results

### Symptom types of pepper anthracnose caused by *Colletotrichum* species

**Figure 1:**
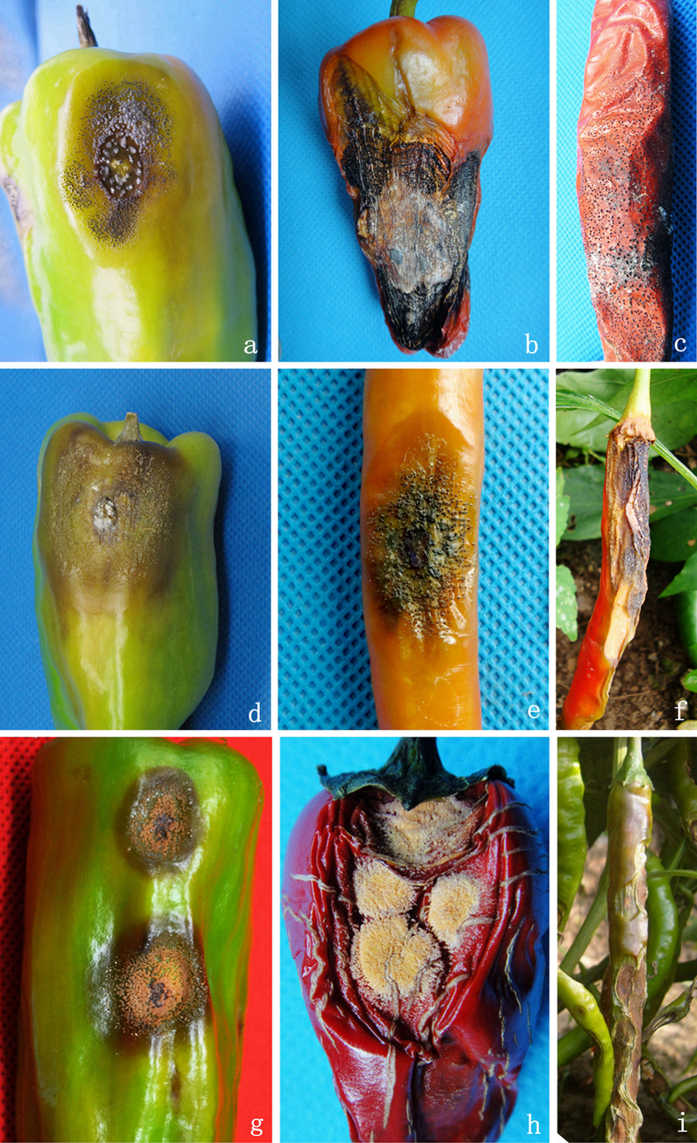
Typical symptoms of pepper anthracnose in the field. **(a–c)** Type I symptoms were characterized by dark brown to black, sunken lesions with a slightly raised rim and many black acervuli on the surface, which produced dirty white conidial masses under humid conditions. (**d–f)** Type II symptoms included dark brown to black, sunken lesions with many black acervuli on the surface, which produced flesh pink, viscous conidial masses under humid conditions. (**g–i)** Type III symptoms included brown to light black to dark brown, sunken, lesions with orange conidial masses.

A total of 173 symptomatic samples were collected from primary pepper-producing regions, covering 31 districts in Sichuan Province, China. Based on the morphological characteristics coupled with the microscopic observations, the following three typical symptom types in the infected pepper fruits in the fields were noted (Fig. 1): Type I: the typical symptoms were variation in colour from dark brown to black, sunken lesions and many black acervuli on the surface, which usually produced dirty white conidial masses under humid conditions. In some cultivars with less pulp, these typical conidial masses were infrequently observed in black acervuli (Fig. 1a–c). The conidia responding for this symptom type had the typical falcate spores; Type II: the symptoms included sunken necrotic tissues, ranging in colour from brown to black, with concentric rings of acervuli (Fig. 1d–f). The main Type II symptoms were similar to the main Type I symptoms, except that the acervuli produced viscous, flesh-pink conidial masses under wet conditions and cylindrical to long cylindrical conidia; and Type III: the typical symptoms included light brown to dark brown tissues, and sunken, orange conidial masses that were powdery in a dry environment; in addition, the conidia were fusiform (Fig. 1g–i). Notably, more than one type of disease symptom was often observed in a single pepper fruit in the field.

### *Colletotrichum* species collection

A total of 352 single-spore cultures were isolated from 173 symptomatic samples. Eighty-eight isolates were subsequently selected for further determination on the basis of their origins, colony characteristics and conidial morphologies (Table 1).

### Morphological and cultural characteristics

Eighty-eight isolates were classified into six morphological groups according to morphological and cultural characteristics. **Group 1** included 23 isolates fitting the description of the *C. gloeosporioides* complex, and **Group 2** included 16 isolates fitting the description of *C. fructicola*. In addition, **Group 3** consisted of 32 isolates matching the description of *C. truncatum*, **Group 4** had seven isolates fitting the description of the *C. acutatum* complex, and **Group 5** consisted of six isolates matching the description of *C. brevisporum*. **Group 6** contained four isolates that did not fit the description of any currently known *Colletotrichum* species. **Group 3 (***C. truncatum*) was the predominant group, accounting for 36.4% of the total isolates. A summary of the morphological data for the *Colletotrichum* species in Groups 1–6 is presented in Table 2.

**Figure 2:**
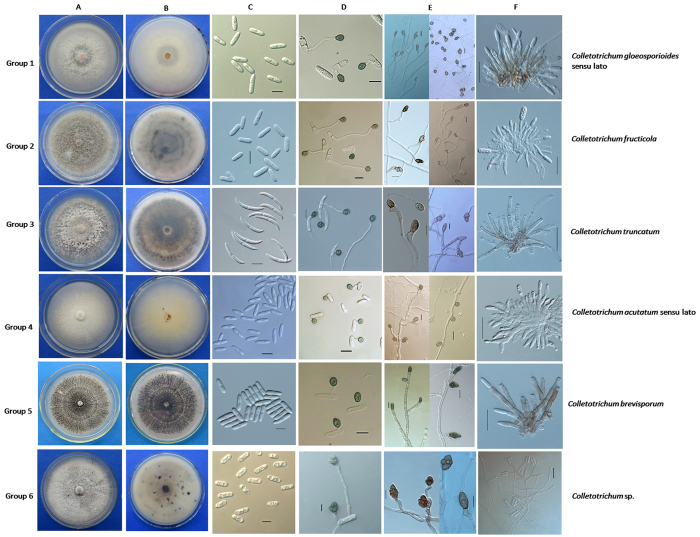
Morphology and cultural characteristics of *Colletotrichum* spp. from pepper anthracnose. (**A**) Upper view of a colony on PDA; (**B**) reverse view of colony on PDA; (**C**) micrographs of conidia of *Colletotrichum* spp.; (**D**) micrographs of conidial appressoria of *Colletotrichum* spp.; (**E**) micrographs of mycelial appressoria of *Colletotrichum* spp.; (**F**) conidiophores. Scale bars = 10 μm for (**C–E**); 20 μm for (**F**).

Colony characteristics (Fig. 2): Distinct morphology on potato dextrose agar (PDA) was observed in each group after 7 days. The isolates from Group 1 produced pale yellowish colonies, with sparse white aerial mycelia. The reverse side of the colonies was white, and many bright orange conidial masses were observed near the inoculum point. The colonies produced by Group 2 isolates varied from white to black-green on PDA, with dense grey aerial mycelia and a few bright orange conidial masses near the inoculum point. The colonies produced by Group 3 isolates varied from pale grey to dark grey, with dense pale grey aerial mycelia and small black granules over the entire surface. The reverse side of the colonies was dark brown, and a few pale yellow conidial masses were observed near the inoculum point. The colonies produced by Group 4 isolates varied from white to pale orange, with dense white aerial mycelia, and the reverse side of the colonies was pale orange. Isolates belonging to Group 5 produced dark grey colonies with sparse grey aerial mycelia. The reverse side of the colonies was grey, and a few bright orange conidial masses were observed near the inoculum point, as well as some spots scattered over the colony surface. Lastly, the isolates from Group 6 produced pale grey colonies, with sparse white aerial mycelia. The colonies from Group 3, 4 and 5 were stable and unique, and the colonies from Group 2 were significantly different compared with those from the other groups under stable culture conditions.

Growth rate (Table 2): Group 4 exhibited a significantly different growth rate compared with the other five groups (P = 0.05). The isolates from Group 6 (6.1 ± 0.4 mm/day) grew the fastest, followed by those from Group 1 (5.6 ± 1.2 mm/day), Group 2 (5.9 ± 0.4 mm/day), Group 5 (5.3 ± 0.6 mm/day), Group 3 (4.5 ± 0.5 mm/day) and Group 4 (3.8 ± 0.4 mm/day).

Conidial morphology (Table 2 and Fig. 2): The following four types of conidia were observed: cylindrical (observed in Groups 1, 2 and 6), falcate (Group 3), fusiform (Group 4) and long cylindrical (Group 5). The conidial widths of Group 6 were significantly different from those of Groups 1 and 2; however, all of these groups had cylindrical conidia with obtuse to slightly rounded ends. The conidia produced by the Group 3 isolates were falcate, with gradual tapering towards each end. Group 4 produced fusiform conidia, whereas Group 5 produced long and cylindrical conidia, with obtuse to slightly rounded ends. The differences in the conidial shapes of Groups 3, 4 and 5 were very significant, allowing these groups to be easily distinguished from one another. Almost all of the conidia were aseptate, but they often developed a septum after germinating and forming appressoria.

Conidial appressorium morphology (Table 2 and Fig. 2): There was little distinction among the groups in terms of the sizes and shapes of conidial appressoria, except for Groups 4 and 6, which exhibited significant differences compared with the other groups. The conidial appressoria of Groups 1, 2, 3 and 5 varied from ovoid to slightly irregular in shape and from brown to dark black in colour. Group 4 produced grey, globular and smaller conidial appressoria. Most of the conidial appressoria produced by Group 6 were irregular and pale brown to dark brown, with a crenate edge.

Mycelial appressorium morphology (Table 2 and Fig. 2): The mycelial appressoria produced by the isolates of Groups 1 and 2 varied from ovoid, clavate and slightly irregular to irregular, smooth or slightly lobed, and they were light brown to brown in colour. The appressoria of Group 3 ranged from ovate, ellipsoidal or slightly irregular to irregular in shape, and they were smooth or lobate and brown to dark brown. The appressoria produced by Group 4 were globose or ovate to slightly irregular, and they were light brown to brown and smaller in size than those of the other groups. In addition, the appressoria produced by Group 5 varied from ovoid, clavate or slightly irregular to irregular in shape. They were smooth or slightly lobed and brown to dark brown and were sometimes black in the middle. Further, the appressoria of Group 6 were ellipsoidal or irregular, smooth or slightly lobed to strongly lobed, solitary or in chains, and light brown to brown in colour.

Conidiophores (Fig. 2): The conidiophores of all groups were hyaline to pale brown, simple or septate, rarely branched, and smooth walled. Four types of conidiophores were observed: (i) nearly cylindrical, but narrower towards the end (as observed in Groups 1 and 2); (ii) cylindrical, with a truncate top (Groups 3 and 5); (iii) shortly clavate, nearly hyaline, with a cylindrical base, and obviously inflated, with gradually tapering towards the top (Group 4); and (iv) frequently produced by mycelia, cylindrical, with swollen ends (oblong) and slight narrowing in some areas (Group 6).

Setae: All isolates from Groups 3 and 5 and some isolates from Group 1 produced setae; in contrast, the isolates from all the other groups rarely produced setae. The setae were commonly smooth, septate, and light brown to dark brown in colour, base cylindrical to conical, and sometimes slightly inflated, and the tips were acute to roundish. No obvious differences in setal characteristics (shape and dimensions) were found among the different groups when grown on PDA.

Sclerotia and Ascomata: Most Group 5 isolates steadily produced a large amount of black solids that appeared similar to sclerotia and were round to irregular and semi-immersed. Conidial masses and setae sometimes formed on the black solids. On PDA, Group 6 isolates always produced ascomata in clusters, which were brown and globose to near globose and possessed a neck. The isolates from the other groups rarely produced ascomata, even in host tissues.

### Phylogenetic analysis

**Figure 3:**
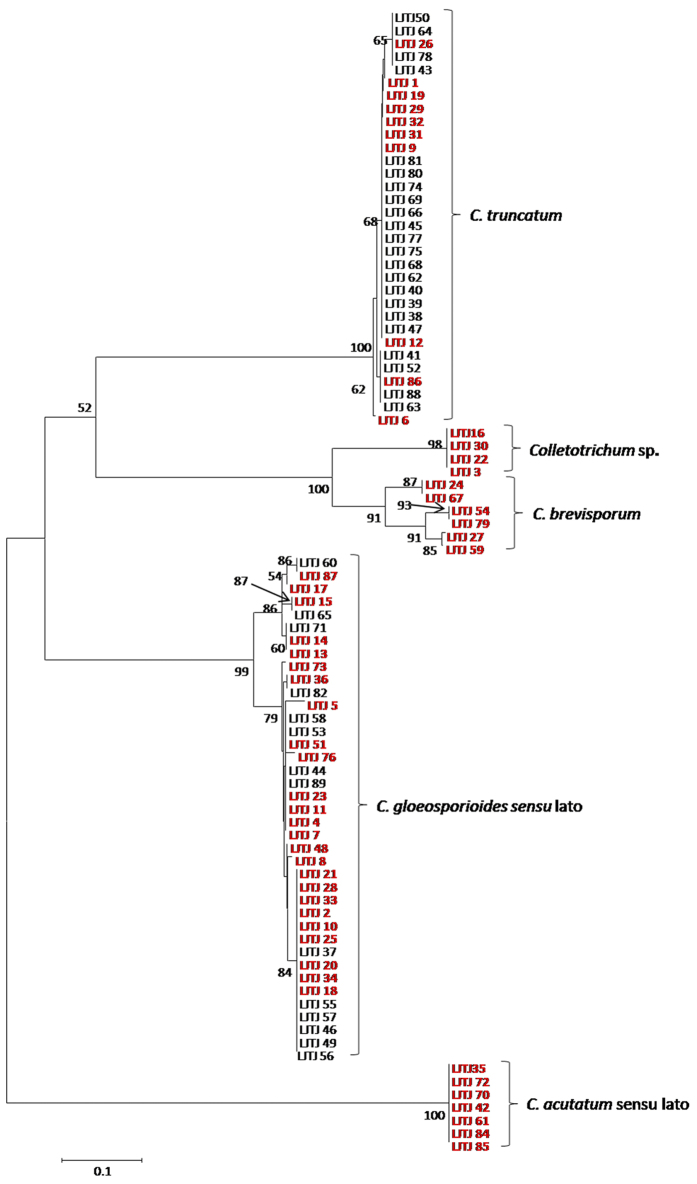
A neighbour-joining tree based on partial GAPDH gene sequences from 88 *Colletotrichum* isolates. Parsimony bootstrap values of more than 50% are shown at the nodes. Isolates selected for subsequent phylogenetic analyses are highlighted in red.

A phylogram generated based on the GAPDH gene region revealed 5 primary clades (i.e., *C. truncatum, C. brevisporum*, *C. gloeosporioides* sensu lato, *C. acutatum* sensu lato and one unknown species (*Colletotrichum* sp.)) (Fig. 3).

Fifty-two representative isolates were chosen from the morphological groups for molecular analysis, including 25 isolates from the *C. gloeosporioides* complex, 10 *C. truncatum* isolates, seven *C. acutatum* sensu lato isolates, six *C. brevisporum* isolates and four *Colletotrichum* sp. isolates.

**Figure 4:**
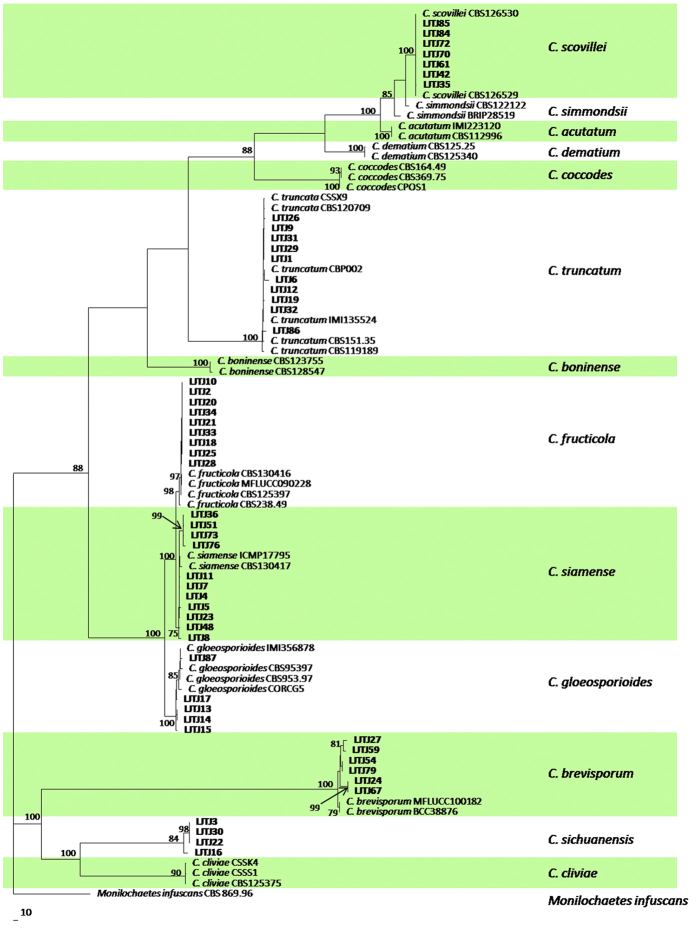
Phylogram generated from maximum parsimony analysis based on alignment of ITS, TUB2, ACT, GADPH and CAL gene sequences, showing the phylogenetic relationships of *Colletotrichum* species causing anthracnose disease in *Capsicum annuum* from Sichuan Province, China. Parsimony bootstrap values of more than 50% are shown at the nodes. Isolates from this study are shown in bold. The tree is rooted with *Monilochaetes infuscans.*

Multi-locus phylogenetic analysis was conducted among 87 strains, with *Monilochaetes infuscans* (CBS 869.96) used as an outgroup (Table 3). The dataset for five genes (ITS, TUB2, ACT, GAPDH and CAL) contained 2,155 characters, including alignment gaps, of which 997 characters were parsimony-informative, 321 were parsimony-uninformative, and 837 were constant. This parsimony analysis resulted in the most parsimonious tree (TL = 2800, CI = 0.7257, RI = 0.9541, RC = 0.6924, and HI = 0.2743). The phylogram showed that the 52 pepper anthracnose isolates belonged to seven distinct clades. The isolates from Group 2 clustered with *C. fructicola*, those from Group 3 clustered with *C. truncatum*, those from Group 4 clustered with *C. scovillei*, and those from Group 5 clustered with *C. brevisporum*. The Group 1 isolates grouped with two clades; 4 isolates clustered with *C. gloeosporioides*, and the remaining isolates clustered with *C. siamense* (Fig. 4). After combining two phylograms (Figs 3 and 4), 8 and 16 strains were found to belong to *C. gloeosporioides* sensu stricto and *C. siamense*, respectively. The isolates from Group 6 were from an unknown species (*Colletotrichum* sp.). The submission number of the sequence alignment in TreeBASE is 18832.

### Taxonomy

**Figure 5:**
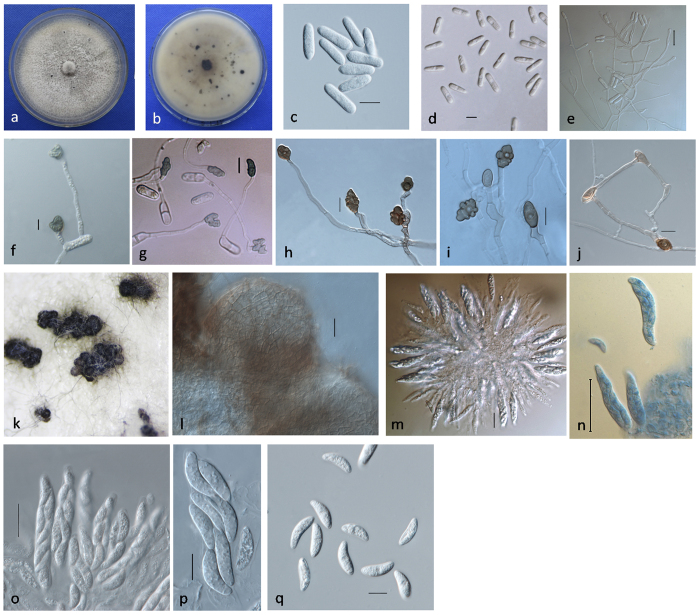
*Colletotrichum sichuanensis* (from holotype). (**a,b**) Colonies on PDA at 7 days, upper (**a**) and reverse (**b**); (**c**,d) conidia; (**e**) conidiogenous cells; (**f,g**) conidial appressoria; (**h–j**) mycelial appressoria; (**k**) ascomata on PDA; (**l**) peridium; (**m–o**) asci; (**p,q**) ascospores. Scale bars: c, d, f–j, p, q = 10 μm; e, l, m, o* = *20 μm; n = 40 μm

*Colletotrichum sichuanensis* G.S. Gong & F.L. Liu, **sp. nov.** (Fig. 5).

MycoBank: MB 815288.

*Etymology*: *sichuanensis*, in reference to the province where the type was found.

Description: Colonies on PDA at first white, later becoming pale grey and reverse pale grey, with a maximum diameter of 68.7 mm over 5 days at 28 °C and a growth rate of 6.1–6.4 mm/day (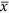 = 6.3 ± 0.1, n = 5). Aerial mycelium white and sparse, with the frequent absence of conidial masses. Ascomata nearly always present in clusters on PDA. Conidiophores generated from mycelia are nearly hyaline, branched, and cylindrical, with slightly swollen ends, simple or occasionally branched. Conidia common on mycelia, one-celled, smooth-walled, hyaline, and cylindrical, with obtuse to slightly rounded ends, 15.0–18.9 × 5.4–6.5 μm (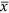 = 16.9 ± 1.0 × 6.2 ± 0.3, n = 30). Conidial appressoria light brown to dark brown, slightly irregular to irregular, crenate or lobed, 8.1–12.4 × 5.4–8.8 μm (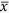 = 10.2 ± 1.0 × 6.8 ± 0.8, n = 30). Appressoria in slide culture light brown to brown, ellipsoidal or irregular, smooth or slightly lobed to strongly lobed, solitary or in chains, 6.4–20.2 × 4.8–9.8 μm (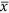 = 11.5 ± 3.0 × 7.0 ± 1.1, n = 30). Setae absent.

Teleomorph: Glomerella sp.

*Ascomata*, light brown to brown, globose to subglobose, with a neck, arranged in clusters. *Peridium* of *textura angularis*, thick-walled. *Asci* 30.8–61.6 × 7.4–13.8 μm (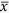 = 47.5 ± 8.0 × 9.5 ± 1.5, n = 30), unitunicate, thin-walled, and clavate. *Ascospores* 10.2–23.3 × 3.9–6.8 μm (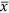 = 17.5 ± 2.6 × 5.4 ± 0.8, n = 30), one-celled, hyaline, and slightly curved to curved, with obtuse to slightly rounded ends.

*Holotype*: Baoxing, Yaan City, Sichuan Province, China, on fruit of *Capsicum annuum*, 5 *September* 2013, coll. G. S. Gong (holotype living culture LJTJ30). A living culture (strain LJTJ30) was deposited at the Department of Plant Pathology of Sichuan Agricultural University. Known distribution: Sichuan Province, China.

Additional examined specimens: Jiangyou, Mianyang City, Sichuan Province, China, on *Capsicum annuum* fruit, 26 July 2013, coll. G. S. Gong (holotype living culture LJTJ3); Yuechi, Guangan City, Sichuan Province, China, on *Capsicum annuum* fruit, 27 August 2013, coll. F. L. Liu (holotype living culture LJTJ16); and Wenjiang, Chengdu City, Sichuan Province, China, on *Capsicum annuum* fruit, 3 July 2013, coll. F. L. Liu (holotype living culture LJTJ22). A living culture (strain LJTJ3, LJTJ16 and LJTJ22) was deposited at the Department of Plant Pathology at Sichuan Agricultural University.

### Pathogenicity tests

**Figure 6:**
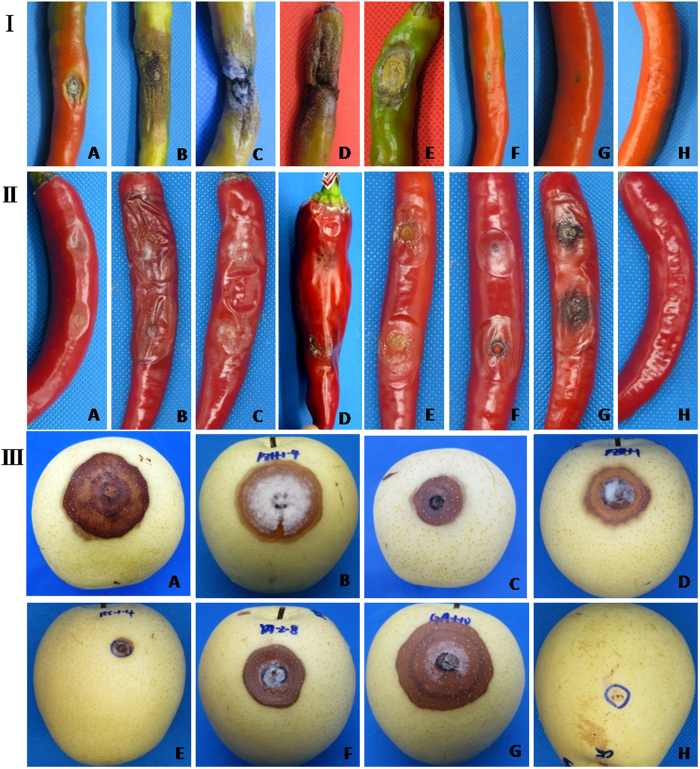
Symptoms in pepper and pear after inoculation with *Colletotrichum* spp. **I**, Symptoms in pepper (*Capsicum annuum* var. *dactylus* M); **II**, symptoms in pepper (*Capsicum annuum* L. var. *conoides* (Mill.) Irish); **III**, symptoms in pear (*Pyrus pyrifolia*); (A), symptoms in pepper and pear inoculated with a mycelial disc of *C. gloeosporioides*; (B), symptoms in pepper and pear inoculated with a mycelial disc of *C. siamense*; (C), symptoms in pepper and pear inoculated with a mycelial disc of *C. fructicola*; (D), symptoms in pepper and pear inoculated with a mycelial disc of *C. truncatum*; (E), symptoms in pepper and pear inoculated with a mycelial disc of *C. scovillei*; (F), symptoms in pepper and pear inoculated with a mycelial disc of *C. brevisporum*; (G), symptoms in pepper and pear inoculated with a mycelial disc of *C. sichuanensis*; (H), the control, inoculated with an agar disc.

Fifty-two representative isolates selected from among the species were used for pathogenicity testing. All of these isolates were pathogenic to both pepper fruits and pears, although the pathogenicity of each species differed across experimental varieties, with different infection incidences. All species were able to infect *Capsicum annuum* L. var. *conoides* (Mill.) Irish and *Pyrus pyrifolia* at a high incidence. However, *C. brevisporum* and *C. sichuanensis* appeared to be only slightly virulent towards *Ca. annuum* var. *dactylus* M, with a rather low infection incidence (Table 4 and Fig. 6). These results indicated that some pepper varieties might be resistant to some *Colletotrichum* species.

Based on the description of the symptoms in pepper after inoculation, *C. truncatum* was determined to be the pathogen causing Type I symptom, characterized by copious black acervuli with seta and dirty white conidial masses produced on decaying tissues under humid conditions (Fig. 1a–c). *C. scovillei* induced Type III symptoms (Fig. 1g–i), and the other species caused Type II symptoms (Fig. 1d–f). Our results indicate that with the exception of *C. truncatum* and *C. scovillei*, it is difficult to differentiate among *Colletotrichum* species based solely on the symptom types in the field.

## Discussion

The primary objective of this study was to identify the *Colletotrichum* species that are currently causing anthracnose disease in pepper grown in Sichuan Province, China. Based on the morphological characteristics and phylogenetic analysis, 88 isolates were identified as *C. gloeosporioides* sensu stricto (eight strains, 9.1%), *C. siamense* (16 strains, 18.2%), *C. fructicola* (15 strains, 17.0%), *C. truncatum* (32 strains, 36.4%), *C. scovillei* (seven strains, 8.0%), *C. brevisporum* (six strains, 6.8%) and *C. sichuanensis* (a new species, four strains, 4.5%). Additionally, *C. gloeosporioides* and *C. siamense* could only be distinguished by phylogenetic analyses and not by morphological analyses. The morphological groupings based on colony characteristics, growth rate, conidial morphology, conidial appressorium morphology and mycelial appressorium morphology were almost completely consistent with the results of phylogenetic analysis derived from the molecular data.

*In vitro* culture-related characteristics were important for differentiating among *Colletotrichum* species^26^. *C. truncatum*, *C. scovillei*, *C. brevisporum*, *C. sichuanensis* isolates and some *C. fructicola* isolates with unique and relatively stable colonies could be easily distinguished. However, the colonies of *C. gloeosporioides*, *C. siamense* and some *C. fructicola* isolates overlapped in terms of their morphological characteristics, and phenotypic variations were identified among the species under different environmental conditions. The colony growth rate of *C. scovillei* was significantly slower than those of the species in the other groups. Previous studies have shown that *C. acutatum* can be differentiated from *C. gloeosporioides* based on its slower growth rate^30^. Than *et al*.^2^ have also suggested that colony growth rates are important for distinguishing among *C. gloeosporioides*, *C. truncatum* and *C. acutatum*. In the present study, the slow growth of *C. scovillei* conformed to the characteristics of the *C. acutatum* complex. The observed differences in conidial size were significant, with the exception of the lengths and widths of Groups 1 and 2. Denoyes and Baudry^31^ used conidial shape to differentiate among *Colletotrichum* species that are pathogenic to strawberries, although Cai *et al*.^25^ and Crouch *et al*.^32^ have suggested that conidial appressoria are taxonomically uninformative and of little use for species identification. In contrast, the conidial appressoria of *C. scovillei* could be easily distinguished from those of the other species examined in our study, in agreement with the results of Du *et al*.^33^. Similarly, Crouch *et al*.^32^ have found that the shapes and sizes of mycelial appressoria in combination with the host range are useful for identifying grass-associated *Colletotrichum* species. We found that the mycelial appressoria produced by *C. scovillei* and *C. brevisporum* were typically smoother than those produced by the other species and that all *C. truncatum* and *C. brevisporum* isolates steadily produced setae. In addition, *C. gloeosporioides* has been reported to produce setae occasionally or under certain conditions^34^, and many other *Colletotrichum* species are known to produce setae^3^. In the present study, the cultural characteristics, colony growth rate, conidial shapes and sizes, and conidial and mycelial appressoria were the primary features used for classification.

Morphological examination was conducted to classify the 88 isolates into six groups, although our multi-locus phylogenetic analysis actually identified seven *Colletotrichum* species. Groups 2–6 contained different *Colletotrichum* species, and Group 1 consisted of two species: *C. gloeosporioides* and *C. siamense*. Thus, morphological criteria alone are not always sufficient for species identification^14^. Indeed, multi-locus phylogeny showed that the isolates with similar morphological characteristics belonged to the *C. gloeosporioides*, *C. siamense* and *C. fructicola* clades. Moreover, the *C. gloeosporioides* and *C. siamense* isolates could not be distinguished according to their morphological and cultural characteristics, indicating that multi-locus phylogenetic analysis is useful for differentiating among species in the *Colletotrichum* genus. Many investigators have suggested the use of multi-locus phylogenetic analysis to overcome the inadequacies of morphological criteria^3^,^17^,^24^,^26^,^27^,^35^,^36^,^37^,^38^,^39^.

*Colletotrichum gloeosporioides* was first described in citrus from Italy^40^. The name *C. gloeosporioides* represents both *C. gloeosporioides* sensu lato, which encompasses the entire species complex, and *C. gloeosporioides* sensu stricto^18^. *C. gloeosporioides* sensu lato consists of at least 22 species, including *C. gloeosporioides*, *C. siamense*, and *C. fructicola*^1^,^18^,^25^,^26^,^41^. *C. siamense* and *C. fructicola* were originally known as opportunistic pathogens of *Coffea arabica* berries in Thailand^26^, and both of these species are non-host-specific. *C. fructicola* has also been reported to be a pathogen causing pepper anthracnose in Thailand^16^, India^17^ and China^7^. Although Than *et al*.^2^ first isolated *C. siamense* from chilli pepper in Thailand, the isolates belonging to *C. siamense* were identified as *C. gloeosporioides* in that study, and Weir *et al*.^18^ later revised the classification. *C. siamense* has also been isolated from pepper in India. However, this species has not been reported to be a causative agent of pepper anthracnose in China. Therefore, this work is the first report of pepper anthracnose caused by *C. siamense*.

*Colletotrichum truncatum*, originally described on *Phaseolus lunatus*, was typified by Damm *et al*.^3^, and this species has been associated with anthracnose on legume crops and pepper, as well as on many other hosts^3^,^9^,^34^. The *C. capsici* isolate typified by Shenoy *et al*.^42^ causes anthracnose in a wide range of hosts, including pepper and legume species^1^,^43^,^44^, and Damm *et al*.^3^ synonymized the *C. capsici* taxon with *C. truncatum* on the basis of its multi-locus phylogeny and morphology. Regardless, not all researchers are in agreement with this viewpoint^1^.

*Colletotrichum acutatum* is widely known as a fruit rot pathogen in strawberry^2^, apple^45^, pepper^2^,^11^ and grape^46^, and this fungus was first recorded in Australia on *Carica papaya*, *Capsicum frutescens* and *Delphinium ajacis* by Simmonds^30^. *C. acutatum* is also a species complex containing at least 14 species, including *C. scovillei*^47^. The ex-type strain of *C. scovillei* was initially identified as *C. acutatum*^48^, and Than *et al*.^2^ also identified *C. scovillei* as *C. acutatum* on chilli pepper from Thailand. Although *C. scovillei* was identified as *C. acutatum* in these two papers, it was later revised by Damm *et al*.^47^. Kanto *et al*.^21^ also isolated *C. scovillei* from sweet pepper in Japan. In our study, we only isolated *C. scovillei* belonging to *C. acutatum* sensu lato from the pepper fruits. Thus, the main species from the *C. acutatum* complex that is pathogenic to pepper in Sichuan Province might be *C. scovillei* rather than *C. acutatum* sensu stricto. To our knowledge, this work is also the first report of *C. scovillei* as a causative agent of pepper anthracnose in China.

*Colletotrichum brevisporum* has been recorded on *Neoregelia* sp. from Thailand, as well as on papaya fruits and *Pandanus pygmaeus* Thouars^35^,^49^. Yang^7^ have also reported *C. brevisporum* on pepper from China. The conidial lengths of *C. brevisporum* in the present study were longer than those reported by Noireung *et al*.^35^, but they were consistent with those reported by Yang^7^.

The results of our phylogenetic analysis strongly support the *Colletotrichum sichuanensis* clade, which is closely related to *C. cliviae*. These two species have similar conidial shapes but different conidial sizes; *C. sichuanensis* has shorter conidia than *C. cliviae* (21.8 μm), with a mean length of 16.7 μm. *C. sichuanensis* also differs from *C. cliviae* with regard to colony colour. In addition, *C. sichuanensis* steadily produced ascomata on PDA, whereas the other species rarely produced ascomata. Further, *C. sichuanensis* grew more slowly in culture than *C. cliviae* (11.3–12.9 mm/day for *C. sichuanensis* compared with 15.2–16 mm/day for *C. cliviae*).

Given that they could infect not only *Capsicum* spp. but also *Pyrus pyrifolia*, all of the species isolated from pepper in our study were non-host-specific. In addition, *C. scovillei* was the most virulent species towards *Capsicum* spp. Tang^6^ found that *C. acutatum* and *C. truncatum* were more virulent than *C. gloeosporioides* and that the *C. acutatum* incubation period was the shortest. Further, Than *et al*.^2^,^14^ reported that *C. acutatum* was a very virulent species that could infect wound-resistant *C. chinense* PBC 932, whereas *C. gloeosporioides* and *C. capsici* (syn. *C. truncatum*) could not.

*Colletotrichum acutatum*^10^, *C. truncatum*^5^ and *C. boninense*^19^ have been previously reported in Sichuan; however, *C. boninense* was not isolated in our study; it is possible that this species was missed during sampling or isolation. In summary, *C. siamense* and *C. scovillei* are recorded for the first time as causing anthracnose in pepper from China. Additionally, we have identified one new species, which has been introduced as *C. sichuanensis*.

## Methods

### Collection and isolation

In 2012 and 2013, pepper fruits with anthracnose symptoms were collected from primary production areas in Sichuan Province, China. Tissues of approximately 5 mm in diameter were collected from the edges of lesions, surface-sterilized with 75% ethanol for 30 s and 1% NaClO for approximately 1 min, washed three times with sterile distilled water, and then dried on sterile filter paper. The treated tissues were plated on PDA supplemented with 50 mg l^−1^ streptomycin. The plates were incubated at 27 ± 1 °C for 5 days. Single-spore cultures were obtained for each *Colletotrichum* isolate according to the procedure described by Gong *et al*.^50^. The resulting strains were maintained on PDA slants at 4 °C for short-term storage and in 25% glycerol at −70 °C for long-term storage.

### Morphological and cultural characterization

Mycelial discs (5 mm diameter) were collected from actively growing areas near the growing edges of 5-day-old cultures, transferred to PDA and incubated at 27 °C in the dark for 10 days. Five replicates were employed. The colony diameter was recorded each day from two perpendicular cross-sections, and the colony characteristics were also recorded.

The sizes and shapes of conidia, asci and ascospores from each culture were recorded. The lengths and widths of 30 conidia, asci and ascospores were measured for each isolate.

Conidial appressoria were induced according to the method of Yang *et al*.^27^.

Mycelial appressoria were produced using an improved slide culture technique, as described by Sutton^51^ and Cai *et al*.^25^. One hundred microlitres of hot water agar (WA) was placed on a sterile slide. Mycelial plugs of approximately 2 mm in diameter were inoculated onto one-third of the WA and then incubated in a Petri dish with wet filter paper at 27 °C. After 5–7 days, agar pieces containing the inoculated plugs were gently removed with a scalpel, and the shapes and sizes of the appressoria that formed along the WA were then recorded.

Samples for microscopy were prepared using clear water or lactic acid and observed with a Carl Zeiss Axio Imager Z2 microscope(Germany) or a Nikon Eclipse 80i microscope(Japan) using differential interference contrast (DIC) illumination.

### DNA extraction

Fifty-two representative isolates were chosen according to the morphological and cultural characteristics and incubated on PDA at 27 °C for 7–10 days. Mycelia were scraped from the colony surface using a sterile medicine spoon. Total genomic DNA was extracted from the isolates using a modified protocol, as outlined by Guo *et al*.^52^.

### PCR amplification and DNA sequencing

As an initial analysis of genetic diversity, the glyceraldehydes-3-phosphate dehydrogenase (GAPDH) gene was amplified from the isolates in this study with the primers GDF/GDR^53^. Fifty-two isolates representing wide ranges of genetic diversity and geographic origins were selected for further investigation.

The nuclear rDNA ITS region and the β-tubulin (TUB2), partial actin (ACT) and calmodulin (CAL) genes were amplified from 52 representative isolates using the primers ITS1/ITS4^54^,^55^, Bt2a/Bt2b^56^, ACT512F/ACT783R^57^ and CL1/CL2A^58^, respectively. PCR was performed under the conditions described by Prihastuti *et al*.^26^.

The amplifications were performed in a 40 μl mixture containing 17 μl ddH_2_O, 20 μl 2 × PCR MasterMix (TIANGEN Co., China), 1 μl DNA template (30–50 ng/μl), and 1 μl of each primer (10 μM). DNA sequencing was performed by Sangon Biotech Co., Ltd. (Shanghai, China).

### Phylogenetic analysis

Alignment of the GAPDH genes of all of the isolates was performed using Clustal X^59^. MEGA v. 5 was used to build a distance tree with the neighbour-joining (NJ) algorithm. The sequences were compared with those in the NCBI sequence database using the BLAST algorithm for approximate identification.

The sequences of the 52 isolates and the reference sequences obtained from GenBank (Table 3) were aligned using Clustal X. Then, a phylogenetic tree was constructed with the combined ITS, TUB2, ACT, GAPDH and CAL dataset.

Parsimony trees were inferred by PAUP v4.0b10 using a heuristic search option with 1,000 random sequence additions^60^. All gaps were treated as missing data. Max trees were unlimited, zero-length branches were collapsed, and all multiple parsimonious trees were saved. Clade stability was assessed by bootstrap (BT) analysis with 1,000 replicates. In addition, descriptive tree statistics, such as parsimony (Tree Length [TL], Consistency Index [CI], Retention Index [RI], Related Consistency Index [RC] and Homoplasy Index [HI]), were calculated.

### Pathogenicity tests

Pears were included in the pathogenicity tests for two main reasons: i) because peppers often are planted in pear orchards; and ii) to assess whether *Colletotrichum* species from pepper are host specific. Fruits of *Capsicum annuum* (*Ca. annuum* var. *dactylus* M and *Ca. annuum* L. var. *conoides* (Mill.) Irish) and *Pyrus pyrifolia* were surface-sterilized in 75% ethanol for 3 min and then rinsed three times in sterile distilled water. The fruits were stabbed lightly with a sterile needle, and a mycelial disc with a diameter of 5 mm from a 4-day-old colony obtained from an isolate grown on PDA at 27 °C was attached to each artificially wounded fruit. The PDA discs were covered with moistened cotton for 3 days. The cotton was then removed, and the fruits were incubated for 14 days in a growth chamber at 27 °C with a 12 h light/12 h dark cycle. Six replicates and an equal number of control fruits inoculated only with agar discs were included.

## Additional Information

**How to cite this article**: Liu, F. *et al*. Molecular and phenotypic characterization of *Colletotrichum* species associated with anthracnose disease in peppers from Sichuan Province, China. *Sci. Rep.* **6**, 32761; doi: 10.1038/srep32761 (2016).

## Figures and Tables

**Table 1:** *Colletotrichum* species isolated from peppers(*Capsicum* spp.) in Sichuan,China.

Species	Isolate no.	Origin	Morphological group	Accession no. (GAPDH)
*Colletotrichum truncatum*	LJTJ1	Chenghua, Chengdu	Group 3	KP823771
*C. fructicola*	LJTJ2	Jiangyou, Mianyang	Group 2	KP823772
*C. sichuanensis*	LJTJ3	Jiangyou, Mianyang	Group 6	KP823773
*C. siamense*	LJTJ4	Jiangyou, Mianyang	Group 1	KP823774
*C. siamense*	LJTJ5	Dong, Panzhihua	Group 1	KP823775
*C. truncatum*	LJTJ6	Dong, Panzhihua	Group 3	KP823776
*C. siamense*	LJTJ7	Dong, Panzhihua	Group 1	KP823777
*C. siamense*	LJTJ8	Dong, Panzhihua	Group 1	KP823778
*C. truncatum*	LJTJ9	Renshou, Meishan	Group 3	KP823779
*C. fructicola*	LJTJ10	Jiangyou, Mianyang	Group 2	KP823780
*C. siamense*	LJTJ11	Dong, Panzhihua	Group 1	KP823781
*C. truncatum*	LJTJ12	Jiangyang, Luzhou	Group 3	KP823782
*C. gloeosporioides*	LJTJ13	Jiangyang, Luzhou	Group 1	KP823783
*C. gloeosporioides*	LJTJ14	Jiangyang, Luzhou	Group 1	KP823784
*C. gloeosporioides*	LJTJ15	Yuechi, Guangan	Group 1	KP823785
*C. sichuanensis*	LJTJ16	Yuechi, Guangan	Group 6	KP823786
*C. gloeosporioides*	LJTJ17	Qianfeng, Guangan	Group 1	KP823787
*C. fructicola*	LJTJ18	Santai, Mianyang	Group 2	KP823788
*C. truncatum*	LJTJ19	Yanting, Mianyang	Group 3	KP823789
*C. fructicola*	LJTJ20	Yanting, Mianyang	Group 2	KP823790
*C. fructicola*	LJTJ21	Yanting, Mianyang	Group 2	KP823791
*C. sichuanensis*	LJTJ22	Wengjiang, Chengdu	Group 6	KP823792
*C. siamense*	LJTJ23	Wengjiang, Chengdu	Group 1	KP823793
*C. brevisporum*	LJTJ24	Xichang, Liangshan	Group 5	KP823794
*C. fructicola*	LJTJ25	Yucheng, Yaan	Group 2	KP823795
*C. truncatum*	LJTJ26	Yucheng, Yaan	Group 3	KP823796
*C. brevisporum*	LJTJ27	Yucheng, Yaan	Group 5	KP823797
*C. fructicola*	LJTJ28	Yucheng, Yaan	Group 2	KP823798
*C. truncatum*	LJTJ29	Baoxing, Yaan	Group 3	KP823799
*C. sichuanensis*	LJTJ30	Baoxing, Yaan	Group 6	KP823800
*C. truncatum*	LJTJ31	Baoxing, Yaan	Group 3	KP823801
*C. truncatum*	LJTJ32	Chenghua, Chengdu	Group 3	KP823802
*C. fructicola*	LJTJ33	Jinjiang, Chengdu	Group 2	KP823803
*C. fructicola*	LJTJ34	Renshou, Meishan	Group 2	KP823804
*C. scovillei*	LJTJ35	Renshou, Meishan	Group 4	KP823805
*C. siamense*	LJTJ36	Pengshan, Meishan	Group 1	KP943522
*C. fructicola*	LJTJ37	Dongpo, Meishan	Group 2	KP943523
*C. truncatum*	LJTJ38	Rongxian, Zigong	Group 3	KP943545
*C. truncatum*	LJTJ39	Guangan, Guangan	Group 3	KP943546
*C. truncatum*	LJTJ40	Pixian, Chengdu	Group 3	KP943547
*C. truncatum*	LJTJ41	Pixian, Chengdu	Group 3	KP943541
*C. scovillei*	LJTJ42	Pujiang, Chengdu	Group 4	KP943516
*C. truncatum*	LJTJ43	Chaotian, Guangyuan	Group 3	KP943548
*C. siamense*	LJTJ44	Dujiangyan, Chengdu	Group 1	KP943531
*C. truncatum*	LJTJ45	Yuechi, Guangan	Group 3	KP943554
*C. fructicola*	LJTJ46	Pujiang, Chengdu	Group 2	KP943525
*C. truncatum*	LJTJ47	Pujiang, Chengdu	Group 3	KP943540
*C. siamense*	LJTJ48	Pengshan, Meishan	Group 1	KP823806
*C. fructicola*	LJTJ49	Hongya, Meishan	Group 2	KP943526
*C. truncatum*	LJTJ50	Hongya, Meishan	Group 3	KP943555
*C. siamense*	LJTJ51	Dujiangyan, Chengdu	Group 1	KP943532
*C. truncatum*	LJTJ52	Jiangyou, Mianyang	Group 3	KP943542
*C. siamense*	LJTJ53	Shuangliu, Chengdu	Group 1	KP943533
*C. brevisporum*	LJTJ54	Shuangliu, Chengdu	Group 5	KP943511
*C. fructicola*	LJTJ55	Shuangliu, Chengdu	Group 2	KP943534
*C. fructicola*	LJTJ56	Shuangliu, Chengdu	Group 2	KP943527
*C. fructicola*	LJTJ57	Wengjiang, Chengdu	Group 2	KP943535
*C. siamense*	LJTJ58	Xinjing, Chengdu	Group 1	KP943536
*C. brevisporum*	LJTJ59	Yucheng, Yaan	Group 5	KP943513
*C. gloeosporioides*	LJTJ60	Yanjiang, Ziyang	Group 1	KP943528
*C. scovillei*	LJTJ61	Chenghua, Chengdu	Group 4	KP943517
*C. truncatum*	LJTJ62	Jinjiang, Chengdu	Group 3	KP943549
*C. truncatum*	LJTJ63	Jinjiang, Chengdu	Group 3	KP943543
*C. truncatum*	LJTJ64	Yuechi, Guangan	Group 3	KP943556
*C. gloeosporioides*	LJTJ65	Yuechi, Guangan	Group 1	KP943529
*C. truncatum*	LJTJ66	Yuechi, Guangan	Group 3	KP943557
*C. brevisporum*	LJTJ67	Qianfeng, Guangan	Group 5	KP943512
*C. truncatum*	LJTJ68	Qianfeng, Guangan	Group 3	KP943550
*C. truncatum*	LJTJ69	Longquanyi, Chengdu	Group 3	KP943558
*C. scovillei*	LJTJ70	Longquanyi, Chengdu	Group 4	KP943515
*C. gloeosporioides*	LJTJ71	Yanting, Mianyang	Group 1	KP943530
*C. scovillei*	LJTJ72	Yanting, Mianyang	Group 4	KP943514
*C. siamense*	LJTJ73	DongQu, Panzhihua	Group 1	KP943537
*C. truncatum*	LJTJ74	Wengjiang, Chengdu	Group 3	KP943559
*C. truncatum*	LJTJ75	Wengjiang, Chengdu	Group 3	KP943551
*C. siamense*	LJTJ76	Wengjiang, Chengdu	Group 1	KP943538
*C. truncatum*	LJTJ77	Wengjiang, Chengdu	Group 3	KP943552
*C. truncatum*	LJTJ78	Wengjiang, Chengdu	Group 3	KP943553
*C. brevisporum*	LJTJ79	Wengjiang, Chengdu	Group 5	KP943510
*C. truncatum*	LJTJ80	Wengjiang, Chengdu	Group 3	KP943560
*C. truncatum*	LJTJ81	Wengjiang, Chengdu	Group 3	KP943561
*C. siamense*	LJTJ82	Wengjiang, Chengdu	Group 1	KP943520
*C. scovillei*	LJTJ84	Xichang, Liangshan	Group 4	KP943519
*C. scovillei*	LJTJ85	Yucheng, Yaan	Group 4	KP943539
*C. truncatum*	LJTJ86	Yucheng, Yaan	Group 3	KP943521
*C. gloeosporioides*	LJTJ87	Yucheng, Yaan	Group 1	KP943544
*C. truncatum*	LJTJ88	Yucheng, Yaan	Group 3	KP943524
*C. siamense*	LJTJ89	Wenjiang, Chengdu	Group 1	KP943518

**Table 2:** Summary of morphological data for *Colletotrichum* isolates.

Group	Species	Colonies appearance	Growth rate (mm/day)	Conidia			Conidial appressoria			Characteristics of mycelial appressoria
				Length (μm)	Width (μm)	Shape	Length (μm)	Width (μm)	Characteristics	
Group 1(23)†	*Colletotrichum gloeosporioides, C. siamense*	pale yellowish colonies, reverse white	5.6 ± 1.2 ab* 4.7–6.7	16.5 ± 2.0 c* 12.0–24.2	5.5 ± 0.6 b* 3.8–7.3	Cylindrical	7.6 ± 1.0 c* 5.5–9.9	5.9 ± 0.6 b* 4.8–7.7	brown to dark black, ovoid to slightly irregular	light brown to brown, ovoid or slightly irregular to irregular
Group 2(16)	*C. fructicola*	white to black green	5.9 ± 0.4 **ab** 5.4–6.6	16.2 ± 2.1 **c** 11.1–25	5.5 ± 0.6 **b** 2.5–7.7	Cylindrical	7.9 ± 0.6 **bc** 6.2–9.1	6.1 ± 0.5 **ab** 4.4–7.2	brown to dark black, ovoid to slightly irregular	light brown to brown, ovoid, clavate and slightly irregular to irregular, smooth or slightly lobed
Group 3(32)	*C. truncatum*	pale grey to dark grey, reverse dark brown	4.5 ± 0.5 **b** 3.9–5.4	24.1 ± 1.9 **a** 18.8–29.87	3.7 ± 0.3 **d** 2.7–4.9	Falcate	7.8 ± 1 **bc** 4.6–11.2	5.4 ± 0.5 **c** 4.1–7	brown to dark black, ovoid to slightly irregular	brown to dark brown, ovate, ellipsoidal or slightly irregular to irregular
Group 4(7)	*C. scovillei*	white to pale orange, reverse pale orange	3.8 ± 0.4 **c** 3.3–4.2	11.7 ± 2.3 **d** 8.2–17.2	3.7 ± 0.3 **d** 3.1–4.6	Fusiform	6.0 ± 1.2 **d** 4.2–9	4.8 ± 0.5 **d** 3.5–6.0	Grey, globular in shape	light brown to brown, globose, ovate to slightly irregular
Group 5(6)	*C. brevisporum*	pale grey, reverse black	5.3 ± 0.6 **b** 5.0–5.8	19.3 ± 1.3 **b** 16.3–22.2	5.2 ± 0.5 **c** 4.2–6.3	Long cylindrical	8.0 ± 0.8 **b** 6.1–9.5	6.2 ± 0.5 **a** 5–7.9	brown to dark black, ovoid to slightly irregular	brown to dark brown, sometimes black in the middle, ovoid or slightly irregular to irregular
Group 6(4)	*C. sichuanensis*	pale grey, reverse pale grey	6.1 ± 0.4 **a** 5.5–6.7	16.7 ± 1.2 **c** 14.2–19.1	6.3 ± 0.4**a** 5.4–6.7	Cylindrical	11.1 ± 1.7 **a** 8–14.2	6.3 ± 2.0 **a** 4.3–11.0	brown to dark brown, irregular with a crenate edge	light brown to brown, ovoid or slightly irregular to irregular

^†^The numbers shown in parentheses represent the number of isolates in each group.

^*^The mean difference is significant at the 0.05 level; the values with same letter in a column do not significantly differ according to Duncan’s multiple range test.

**Table 3:** Details of the *Colletotrichum* isolates used in this study, including the hosts, locations and GenBank accession numbers of the generated sequences.

Species	Strain no.	Host	Location	GenBank accession number				
				ITS	TUB2	ACT	GPDH	CAL
*Colletotrichum acutatum*	BRIP 28519	*Carica papaya*	Australia	FJ 972601	FJ 907443	FJ 907428	FJ 972580	FJ 917510
*C. acutatum*	CBS 29467	*Carica papaya*	Australia	FJ 972610	FJ 907444	FJ 907429	FJ 972581	FJ 917511
*C. boninense*	CBS 128547	*Camellia* sp.	New Zealand	JQ005159	JQ005593	JQ005507	JQ005246	JQ005680
*C. boninense*	CBS 123755	*Crinum asiaticum*	Japan	JQ005153	JQ005588	JQ005501	JQ005240	JQ005674
*C. brevisporum*	BCC 38876*	*Neoregelia* sp.	Thailand	JN050238	JN050244	JN050216	JN050227	JN050222
*C. brevisporum*	MFLUCC100182	*Pandanus pygmaeus*	Thailand	JN050239	JN050245	JN050217	JN050228	—
***C. brevisporum***	**LJTJ24**	***Capsicum*****sp.**	**China**	**KP748215**	—	**KP823736**	**KP823794**	—
***C. brevisporum***	**LJTJ27**	***Capsicum*****sp.**	**China**	**KP748218**	—	**KP823737**	**KP823797**	—
***C. brevisporum***	**LJTJ54**	***Capsicum*****sp.**	**China**	**KP943578**	—	**KP943568**	**KP943511**	—
***C. brevisporum***	**LJTJ59**	***Capsicum*****sp.**	**China**	**KP943579**	—	**KP943569**	**KP943513**	—
***C. brevisporum***	**LJTJ67**	***Capsicum*** **sp.**	**China**	**KP943580**	—	**KP943570**	**KP943512**	—
***C. brevisporum***	**LJTJ79**	***Capsicum*****sp.**	**China**	**KP943581**	—	**KP943571**	**KP943510**	—
*C. cliviae*	CBS 125375*	*Clivia miniata*	China	JX519223	JX519249	JX519240	JX546611	—
*C. cliviae*	CSSK4	*Clivia miniata*	China	GQ485607	GQ849440	GQ856777	GQ856756	GQ849464
*C. cliviae*	CSSS1	*Clivia miniata*	China	GU109479	GU085869	GU085861	GU085868	GU085864
*C. coccodes*	CBS 164.49	*Solanum tuberosum*	Netherlands	HM171678	—	HM171666	HM171672	HM171669
*C. coccodes*	CBS 369.75*	*Solanum tuberosum*	Netherlands	HM171679	—	HM171667	HM171673	HM171670
*C. coccodes*	CPOS1	*Solanum tuberosum*	China	GQ485588	GQ849444	GQ856787	GQ856744	HM171670
*C. dematium*	CBS 125.25*	*Eryngium campestre*, dead leaf	France	GU227819	GU228113	GU227917	GU228211	—
*C. dematium*	CBS 125340	*Apiaceae*, dead stem	Czech Rep	GU227820	GU228114	GU227918	GU228212	—
*C. fructicola*	ICMP 18581*, CBS 130416	*Coffea arabica*	Thailand	JX010165	JX010405	FJ907426	JX010033	FJ917508
*C. fructicola*	MFLUCC090228*	*Coffea arabica*	Thailand	FJ972603	FJ907441	FJ907426	FJ972578	FJ917508
*C. fructicola*	CBS 125397*, ICMP 18646	*Tetragastris panamensis*	Panama	JX010173	JX010409	JX009581	JX010032	JX009674
*C. fructicola*	CBS 238.49, ICMP 17921	*Ficus habrophylla*	Germany	JX010181	JX010400	JX009495	JX009923	JX009671
***C. fructicola***	**LJTJ2**	***Capsicum*****sp.**	**China**	**KP748192**	**KP823854**	**KP823742**	**KP823772**	**KP823812**
***C. fructicola***	**LJTJ10**	***Capsicum*****sp.**	**China**	**KP748201**	**KP823855**	**KP823743**	**KP823780**	**KP823813**
***C. fructicola***	**LJTJ18**	***Capsicum*****sp.**	**China**	**KP748209**	**KP823856**	**KP823744**	**KP823788**	**KP823814**
***C. fructicola***	**LJTJ20**	***Capsicum*****sp.**	**China**	**KP748211**	**KP823857**	**KP823745**	**KP823790**	**KP823815**
***C. fructicola***	**LJTJ21**	***Capsicum*****sp.**	**China**	**KP748212**	**KP823858**	**KP823746**	**KP823791**	**KP823816**
***C. fructicola***	**LJTJ25**	***Capsicum*****sp.**	**China**	**KP748216**	**KP823859**	**KP823747**	**KP823795**	**KP823817**
***C. fructicola***	**LJTJ28**	***Capsicum*****sp.**	**China**	**KP748219**	**KP823860**	**KP823748**	**KP823798**	**KP823818**
***C. fructicola***	**LJTJ33**	***Capsicum*****sp.**	**China**	**KP748224**	**KP823861**	**KP823749**	**KP823803**	**KP823819**
***C. fructicola***	**LJTJ34**	***Capsicum*****sp.**	**China**	**KP748225**	**KP823862**	**KP823750**	**KP823804**	**KP823820**
*C. gloeosporioides*	CBS 95397	*Citrus sinensis*	Italy	FJ972609	FJ907445	FJ 907430	FJ 972582	FJ 917512
*C. gloeosporioides*	CBS 953.97*	*Citrus sinensis*	Italy	GQ485605	GQ849434	GQ856782	GQ856762	GQ849452
*C. gloeosporioides*	IMI 356878*	*Citrus sinensis*	Italy	JX010152	JX010445	JX009531	JX010056	JX009731
*C. gloeosporioides*	CORCG5	*Vanda* sp.	China	HM034809	HM034811	HM034801	HM034807	HM034803
***C. gloeosporioides***	**LJTJ13**	***Capsicum*****sp.**	**China**	**KP748204**	**KP823863**	**KP823751**	**KP823783**	**KP823821**
***C. gloeosporioides***	**LJTJ14**	***Capsicum*****sp.**	**China**	**KP748205**	**KP823864**	**KP823752**	**KP823784**	**KP823822**
***C. gloeosporioides***	**LJTJ15**	***Capsicum*****sp.**	**China**	**KP748206**	**KP823865**	**KP823753**	**KP823785**	**KP823823**
***C. gloeosporioides***	**LJTJ17**	***Capsicum*****sp.**	**China**	**KP748208**	**KP823866**	**KP823754**	**KP823787**	**KP823824**
***C. gloeosporioides***	**LJTJ87**	***Capsicum*****sp.**	**China**	**KT936448**	—	**KT936437**	**KP943544**	**KT936431**
*C. scovillei*	CBS 126529*	*Capsicum* sp.	Indonesia	JQ948267	JQ949918	JQ949588	JQ948597	—
*C. scovillei*	CBS 126530	*Capsicum* sp.	Indonesia	JQ948268	JQ949919	JQ949589	JQ948598	—
***C. scovillei***	**LJTJ35**	***Capsicum*****sp.**	**China**	**KP748226**	**KP823849**	**KP823735**	**KP823805**	**KP823807**
***C. scovillei***	**LJTJ42**	***Capsicum*****sp.**	**China**	**KP943572**	**KP943588**	**KP943562**	**KP943516**	**KP943582**
***C. scovillei***	**LJTJ61**	***Capsicum*****sp.**	**China**	**KP943573**	**KP943589**	**KP943563**	**KP943517**	**KP943583**
***C. scovillei***	**LJTJ70**	***Capsicum*****sp.**	**China**	**KP943574**	**KP943590**	**KP943564**	**KP943515**	**KP943584**
***C. scovillei***	**LJTJ72**	***Capsicum*****sp.**	**China**	**KP943575**	**KP943591**	**KP943565**	**KP943514**	**KP943585**
***C. scovillei***	**LJTJ84**	***Capsicum*****sp.**	**China**	**KP943576**	**KP943592**	**KP943566**	**KP943518**	**KP943586**
***C. scovillei***	**LJTJ85**	***Capsicum*****sp.**	**China**	**KP943577**	**KP943593**	**KP943567**	**KP943519**	**KP943587**
*C. siamense*	ICMP 17795	*Malus* x *domestica*	USA	JX010162	JX010393	JX009506	JX010051	JX009703
*C. siamense*	ICMP 18578*, CBS 130417	*Coffea arabica*	Thailand	JX010171	JX010404	FJ907423	JX009924	FJ917505
***C. siamense***	**LJTJ4**	***Capsicum*****sp.**	**China**	**KP748194**	**KP823867**	**KP823755**	**KP823774**	—
***C. siamense***	**LJTJ5**	***Capsicum*****sp.**	**China**	**KP748195**	**KP823868**	**KP823756**	**KP823775**	**KP823825**
***C. siamense***	**LJTJ7**	***Capsicum*****sp.**	**China**	**KP748198**	**KP823869**	**KP823757**	**KP823777**	**KP823826**
***C. siamense***	**LJTJ8**	***Capsicum*****sp.**	**China**	**KP748199**	**KP823870**	**KP823758**	**KP823778**	**KP823827**
***C. siamense***	**LTTJ11**	***Capsicum*****sp.**	**China**	**KP748202**	**KP823871**	**KP823759**	**KP823781**	**KP823828**
***C. siamense***	**LJTJ23**	***Capsicum*****sp.**	**China**	**KP748214**	**KP823872**	**KP823760**	**KP823793**	**KP823829**
***C. siamense***	**LJTJ36**	***Capsicum*****sp.**	**China**	**KT936443**	**KT936438**	**KT936432**	**KP943522**	—
***C. siamense***	**LJTJ48**	***Capsicum*****sp.**	**China**	**KP748227**	**KP823873**	**KP823761**	**KP823806**	**KP823830**
***C. siamense***	**LTTJ51**	***Capsicum*****sp.**	**China**	**KT936444**	**KT936439**	**KT936433**	**KP943532**	**KT936427**
***C. siamense***	**LJTJ73**	***Capsicum*****sp.**	**China**	**KT936445**	**KT936440**	**KT936434**	**KP943537**	**KT936428**
***C. siamense***	**LJTJ76**	***Capsicum*****sp.**	**China**	**KT936446**	**KT936441**	**KT936435**	**KP943538**	**KT936429**
***C. sichuanensis***	**LJTJ3**	***Capsicum*****sp.**	**China**	**KP748193**	**KP823850**	**KP823738**	**KP823773**	**KP823808**
***C. sichuanensis***	**LJTJ16**	***Capsicum*****sp.**	**China**	**KP748207**	**KP823851**	**KP823739**	**KP823786**	**KP823809**
***C. sichuanensis***	**LJTJ22**	***Capsicum*****sp.**	**China**	**KP748213**	**KP823852**	**KP823740**	**KP823792**	**KP823810**
***C. sichuanensis***	**LJTJ30**	***Capsicum*****sp.**	**China**	**KP748221**	**KP823853**	**KP823741**	**KP823800**	**KP823811**
*C. simmondsii*	CBS 122122*	*Carica papaya*, fruit	Australia	JQ948276	JQ949927	JQ949597	JQ948606	—
*C. simmondsii*	BRIP 28519*	*Carica papaya*, fruit	Australia	GQ485606	GQ856784	GQ849430	GQ856763	GQ849454
*C. truncatum*	CBS 151.35*	*Phaseolus lunatus*	USA	GU227862	GU228156	GU227960	GU228254	—
*C. truncatum*	CBP002	*Brassica parachinensis* Bailey	China	KF030677	KF240819	KF158412	KF300886	KF114851
*C. truncatum*	CSSX9	*Hymenocallis americana*	China	GQ485594	GQ849436	GQ856772	GQ856752	GQ849461
*C. truncatum*	CBS 119189	*Phaseolus lunatus*	USA	GU227863	GU228157	GU227961	GU228255	—
*C. truncatum*	IMI 135524	*Clitoria ternatea*	Sudan	GU227874	GU228168	GU227972	GU228266	—
*C. truncatum*	CBS 120709	*Capsicum frutescens*	India	GQ485593	GQ849429	GQ856783	GQ856753	GQ849453
***C. truncatum***	**LJTJ1**	***Capsicum*****sp.**	**China**	**KP748196**	**KP823840**	**KP823762**	**KP823771**	**KP823831**
***C. truncatum***	**LJTJ6**	***Capsicum*****sp.**	**China**	**KP748197**	**KP823841**	**KP823763**	**KP823776**	**KP823832**
***C. truncatum***	**LJTJ9**	***Capsicum*****sp.**	**China**	**KP748200**	**KP823842**	**KP823764**	**KP823779**	**KP823833**
***C. truncatum***	**LJTJ12**	***Capsicum*****sp.**	**China**	**KP748203**	**KP823843**	**KP823765**	**KP823782**	**KP823834**
***C. truncatum***	**LJTJ19**	***Capsicum*****sp.**	**China**	**KP748210**	**KP823844**	**KP823766**	**KP823789**	**KP823835**
***C. truncatum***	**LJTJ26**	***Capsicum*****sp.**	**China**	**KP748217**	**KP823845**	**KP823767**	**KP823796**	**KP823836**
***C. truncatum***	**LJTJ29**	***Capsicum*****sp.**	**China**	**KP748220**	**KP823846**	**KP823768**	**KP823799**	**KP823837**
***C. truncatum***	**LJTJ31**	***Capsicum*****sp.**	**China**	**KP748222**	**KP823847**	**KP823769**	**KP823801**	**KP823838**
***C. truncatum***	**LJTJ32**	***Capsicum*****sp.**	**China**	**KP748223**	**KP823848**	**KP823770**	**KP823802**	**KP823839**
***C. truncatum***	**LJTJ86**	***Capsicum*****sp.**	**China**	**KT936447**	**KT936442**	**KT936436**	**KP943521**	**KT936430**
*Monilochaetes infuscans*	CBS 869.96	Unknown	Unknown	JQ005780	JQ005864	JQ005843	—	—

ITS: rDNA-ITS region; TUB 2: ß-tubulin; ACT: actin; GPDH: glyceraldehyde-3-phosphate dehydrogenase; and CAL: calmodulin. The isolates from this study are indicated in bold letters.

^*^Ex-type cultures.

**Table 4:** Pathogenicity testing of *Colletotrichum* species from *Capsicum* spp.

Species	Mean infection incidence (%)		
	*Capsicum annuum* var. *dactylus* M^a^	*Capsicum annuum* L. var. *conoides* (Mill.) Irish^b^	*Pyrus pyrifolia*^b^
*Colletotrichum gloeosporioides*	54	72	100
*C. siamense*	64	91	100
*C. fructicola*	58	83	100
*C. truncatum*	93	75	90
*C. scovillei*	100	100	67
*C. brevisporum*	8	60	67
*C. sichuanensis*	9	85	90
CK	0	0	0

^a^Disease symptoms were recorded at 14 days after inoculation of *Capsicum annuum* var. *dactylus* M.

^b^Disease symptoms were recorded at 7 days after inoculation of *Capsicum annuum* L. var. *conoides* (Mill.) Irish and *Pyrus pyrifolia*.

## References

[b1] Hyde K. D. . *Colletotrichum*-names in current use. Fungal Divers. 39, 147–182 (2009).

[b2] Than P. P. . Characterization and pathogenicity of *Colletotrichum* species associated with anthracnose on chilli (*capsicum* spp.) in Thailand. Plant Pathol. 57, 562–572 (2008).

[b3] Damm U., Woudenberg J. H. C., Cannon P. F. & Crous P. W. *Colletotrichum* species with curved conidia from herbaceous hosts. Fungal Divers. 39, 45–87 (2009).

[b4] Montri P., Taylor P. W. J. & Mongkolporn O. Pathotypes of *Colletotrichum* capsici, the causal agent of chili anthracnose, in Thailand. Plant Dis. 93, 17–20 (2009). 10.1094/PDIS-93-1-0017 30764264

[b5] Li N. *Study on the species of anthracnose pathogens and the groups genetic diversity*, Master’s thesis (Sichuan Agricultural University, 2012).

[b6] Tang J. M. *A study on pathogens identification of pepper fruit anthracnose and their biological characteristics in Guangxi*, Master’s thesis (Guangxi University, 2012).

[b7] Yang Y. L. *Multi-locus phylogeny of Colletotrichum species in Guizhou, Yunnan and Guangxi, China*. PhD thesis (Huazhong Agricultural University, 2010).

[b8] Anderson J. M., Aitken E. A. B., Dann E. K. & Coates L. M. Morphological and molecular diversity of *Colletotrichum* spp. Causing pepper spot and anthracnose of lychee (*litchi chinensis*) in Australia. Plant Pathol. 62, 279–288 (2013).

[b9] Ramdial H. & Rampersad S. N. Characterization of *Colletotrichum* spp. Causing anthracnose of bell pepper (*Capsicum annuum* L.) in Trinidad. Phytoparasitica 43, 37–49 (2014).

[b10] Xia H., Wang X. L., Zhu H. J. & Gao B. D. First report of anthracnose caused by *Glomerella acutata* on chili pepper in China. Plant Dis. 95, 219 (2011). 10.1094/PDIS-10-10-0727 30743443

[b11] Harp T., Kuhn P., Roberts P. D. & Pernezny K. L. Management and cross-infectivity potential of *Colletotrichum acutatum* causing anthracnose on bell pepper in Florida. Phytoparasitica 42, 31–39 (2014).

[b12] Shin H. J., Xu T., Zhang C. L. & Cheng Z. J. The comparative study of capsicum anthracnose pathogens from Korea with that of China. Journal of Zhejiang University 26, 629–634 (2000).

[b13] Harp T. L. . The etiology of recent pepper anthracnose outbreaks in Florida. Crop Protect. 27, 1380–1384 (2008).

[b14] Than P. P. . Chilli anthracnose disease caused by *Colletotrichum* species. J. Zhejiang U. Sci. 9, B 9, 764–778 (2008). 10.1631/jzus.B0860007 PMC2565739 18837103

[b15] Sharma P. N. . First report on association of *Colletotrichum* coccodes with chili anthracnose in India. Plant Dis. 95, 1584–1584 (2011). 10.1094/PDIS-04-11-0270 30731988

[b16] Phoulivong S. . *Colletotrichum gloeosporioides* is not a common pathogen on tropical fruits. Fungal Divers. 44, 33–43 (2010).

[b17] Sharma G. & Shenoy B. D. *Colletotrichum fructicola* and *C. Siamense* are involved in chilli anthracnose in India. Arch. Phytopathol. Plant Protect. 47, 1179–1194 (2014).

[b18] Weir B. S., Johnston P. R. & Damm U. The *Colletotrichum gloeosporioides* species complex. Stud. Mycol. 73, 115–180 (2012). 23136459 10.3114/sim0011 PMC3458417

[b19] Diao Y. Z., Fan J. R., Wang Z. W. & Liu X. L. First report of *Colletotrichum boninense* causing anthracnose on pepper in China. Plant Dis. 97, 138–138 (2013). 10.1094/PDIS-04-12-0403-PDN 30722274

[b20] Tozze H. J. Jr. . First report of *Colletotrichum boninense* causing anthracnose on pepper in Brazil. Plant Dis. 93, 106–106 (2009). 10.1094/PDIS-93-1-0106A 30764285

[b21] Kanto T. . Anthracnose of sweet pepper caused by *Colletotrichum scovillei* in Japan. J. Gen. Plant Pathol. 80, 73–78 (2014).

[b22] Zhang G. Z. . Identification of pepper anthracnose and resistant screen of breeding materials in Sichuan. Southwest China Journal of Agricultural Sciences 26, 1026–1029 (2013).

[b23] Lima N. B. . Five *Colletotrichum* species are responsible for mango anthracnose in northeastern Brazil. Fungal Divers. 61, 75–88 (2013).

[b24] Tao G. . Endophytic *Colletotrichum* species from *Bletilla ochracea* (Orchidaceae), with descriptions of seven new speices. Fungal Divers. 61, 139–164 (2013).

[b25] Cai L. . A polyphasic approach for studying *Colletotrichum*. Fungal Divers. 39, 183–204 (2009).

[b26] Prihastuti H. . Characterization of *Colletotrichum* species associated with coffee berries in northern Thailand. Fungal Divers. 39, 89 (2009).

[b27] Yang Y. L. . *Colletotrichum* anthracnose of Amaryllidaceae. Fungal Divers. 39, 123–146 (2009).

[b28] Niu X. . *Colletotrichum* species associated with jute (*Corchorus capsularis* L.) anthracnose in southeastern China. Sci. Rep. 6, 25179, 10.1038/srep25179 (2016). 27121760 PMC4848545

[b29] Manamgoda D. S. . Endophytic *Colletotrichum* from tropical grasses with a new species *C. endophytic*a. Fungal Divers. 61, 107–115 (2013).

[b30] Simmonds J. H. A study of the species of *Colletotrichum* causing ripe fruit rots in Queensland. Queensland Journal of Agricultural and Animal Science 22, 437–459 (1965).

[b31] Denoyes B. & Baudry A. Species identification and pathogenicity study of French *Colletotrichum* strains isolated from strawberry using morphological and cultural characteristics. Phytopathology 85, 53–57 (1995).

[b32] Crouch J. A., Clarke B. B., White J. F. & Hillman B. I. Systematic analysis of the falcate-spored graminicolous *Colletotrichum* and a description of six new species from warm-season grasses. Mycologia 101, 717–732 (2009). 19750952 10.3852/08-230

[b33] Du M., Schardl C. L., Nuckles E. M. & Vaillancourt L. J. Using mating-type gene sequences for improved phylogenetic resolution of *Collectotrichum* species complexes. Mycologia 97, 641–658 (2005). 16392253 10.3852/mycologia.97.3.641

[b34] Sawant I. S. . Emergence of *Colletotrichum gloeosporioides* sensu lato as the dominant pathogen of anthracnose disease of grapes in India as evidenced by cultural, morphological and molecular data. Australasian Plant Pathol. 41, 493–504 (2012).

[b35] Noireung P. . Novel species of *Colletotrichum* revealed by morphology and molecular analysis. Cryptogam, Mycol. 33, 347–362 (2012).

[b36] Huang F. . *Colletotrichum* species associated with cultivated citrus in China. Fungal Divers. 61, 61–74 (2013).

[b37] Liu F., Damm U., Cai L. & Crous P. W. Species of the *Colletotrichum gloeosporioides* complex associated with anthracnose diseases of *Proteaceae*. Fungal Divers. 61, 89–105 (2013).

[b38] Udayanga D. . What are the common anthracnose pathogens of tropical fruits? Fungal Divers. 61, 165–179 (2013).

[b39] Vieira W. A. S. . Endophytic species of *Colletotrichum* associated with mango in northeastern Brazil. Fungal Divers. 67, 181–202 (2014).

[b40] Penzig D. O. Funghi agrumicoli. Contribuzione allo studio dei funghi parassiti degli agrumi, Vol. 2, Michelia (1882).

[b41] Rueda-Hernández K. R. . Differential organ distribution, pathogenicity and benomyl sensitivity of *Colletotrichum* spp. From blackberry plants in Northern Colombia. J. Phytopathol. 161, 246–253 (2013).

[b42] Shenoy B. D., Jeewon R. & Lam W. H. Morpho-molecular characterisation and epitypification of *Colletotrichum capsici* (*Glomerellaceae*, *Sordariomycetes*), the causative agent of anthracnose in chilli. Fungal Divers. 27, 197–211 (2007).

[b43] Pring R. J., Nash C., Zakaria M. & Bailey J. A. Infection process and host range of *Colletotrichum capsici*. Physiol. Mol. Plant Pathol. 46, 137–152 (1995).

[b44] Chai A. . Identification of *Colletotrichum capsici* (Syd.) butler causing anthracnose on pumpkin in China. Can. J. Plant Pathol. 36, 121–124 (2014).

[b45] Víchová J., Stanková B. & Pokorný R. First report of *Colletotrichum acutatum* on tomato and apple fruits in the Czech Republic. Plant Dis. 96, 769–769 (2012). 10.1094/PDIS-10-11-0849-PDN 30727569

[b46] Samuelian S. K., Greer L. A., Savocchia S. & Steel C. C. Application of Cabrio (a.i. pyraclostrobin) at flowering and veraison reduces the severity of bitter rot (*Greeneria uvicola*) and ripe rot (*Colletotrichum acutatum*) of grapes. Aust. J. Grape Wine Res. 20, 292–298 (2014).

[b47] Damm U., Cannon P. F., Woudenberg J. H. & Crous P. W. The *Colletotrichum acutatum* species complex. Stud. Mycol. 73, 37–113 (2012). 23136458 10.3114/sim0010 PMC3458416

[b48] Nirenberg H. I., Feiler U. & Hagedorn G. Description of *Colletotrichum lupini* comb. Nov. inmodern terms. Mycologia 94, 307–320 (2002). 21156502

[b49] Vieira W. A. S. . First report of papaya fruit anthracnose caused by *Colletotrichum brevisporum* in Brazil. Plant Dis. 97, 1659 (2013). 10.1094/PDIS-05-13-0520-PDN 30716854

[b50] Gong G. S. . A simple method for single fungal spore isolation. Journal of Maize Sciences 18, 126–127, 134 (2010).

[b51] Sutton B. C. The Coelomycetes. Fungi imperfecti with pycnidia, acervuli and stromata (Commonwealth Mycological Institute, 1980).

[b52] Guo L. D., Hyde K. D. & Liew E. C. Y. Identification of endophytic fungi from *Livistona chinensis* based on morphology and rDNA sequences. New Phytol. 147, 617–630 (2000). 10.1046/j.1469-8137.2000.00716.x 33862946

[b53] Templeton M. D., Rikkerink E. H., Solon S. L. & Crowhurst R. N. Cloning and molecular characterization of the glyceraldehyde-3-phosphate dehydrogenase-encoding gene and cDNA from the plant pathogenic fungus *Glomerella cingulata*. Gene 122, 225–230 (1992). 1452034 10.1016/0378-1119(92)90055-t

[b54] White T. J., Bruns T., Lee S. & Taylor J. Amplification and direct sequencing of fungal ribosomal RNA genes for phylogenetics. PCR Protocols: A Guide to Methods and Applications 18, 315–322 (1990).

[b55] Gardes M. & Bruns T. D. ITS primers with enhanced specificity for basidiomycetes–application to the identification of mycorrhizae and rusts. Mol. Ecol. 2, 113–118 (1993). 8180733 10.1111/j.1365-294x.1993.tb00005.x

[b56] Glass N. L. & Donaldson G. C. Development of primer sets designed for use with the PCR to amplify conserved genes from filamentous ascomycetes. Appl. Environ. Microbiol. 61, 1323–1330 (1995). 7747954 10.1128/aem.61.4.1323-1330.1995 PMC167388

[b57] Carbone I. & Kohn L. M. A method for designing primer sets for speciation studies in filamentous ascomycetes. Mycologia 91, 553–556 (1999).

[b58] O’Donnell K., Nirenberg H. I., Aoki T. & Cigelnik E. A multigene phylogeny of the *Gibberella fujikuroi* species complex: detection of additional phylogenetically distinct species. Mycoscience 41, 61–78 (2000).

[b59] Thompson J. D. . The CLUSTAL_X windows interface: flexible strategies for multiple sequence alignment aided by quality analysis tools. Nucleic Acids Res. 25, 4876–4882 (1997). 9396791 10.1093/nar/25.24.4876 PMC147148

[b60] Swofford. PAUP* Beta10. phylogenetic analysis using parsimony (*and other methods). Version 4b10 (Sinauer Associates, 2002).

